# A Case of Monkeypox Infection in an Unvaccinated HIV-Positive Male in Rural Alabama

**DOI:** 10.7759/cureus.31383

**Published:** 2022-11-11

**Authors:** Kristine Wong, Malvika Chaudhary, Raul Magadia

**Affiliations:** 1 Infectious Disease, Alabama College of Osteopathic Medicine, Anniston, USA; 2 Infectious Disease, Northeast Alabama Regional Medical Center, Anniston, USA

**Keywords:** mpxv, mpox, hiv, monkeypox virus, case report, monkeypox

## Abstract

Non-endemic cases of monkeypox infection have been increasingly reported worldwide since early May 2022. Here we report a case of self-limited monkeypox disease in an unvaccinated male who was an HIV patient but did not require treatment with tecovirimat. This is the first reported case of monkeypox in Calhoun County, Alabama.

## Introduction

Monkeypox is a viral zoonosis caused by the monkeypox virus, an enveloped double-stranded DNA virus of the Orthopoxvirus genus in the family Poxviridae [1]. Sporadic endemic outbreaks of monkeypox have been reported in Africa; however, more than 3000 cases have been reported in over 50 countries since early May 2022, prompting greater scrutiny of this virus by the World Health Organization (WHO) as a potential public health concern [2]. Human-to-human transmission of monkeypox results from prolonged close contact with skin lesions, respiratory secretions, or contaminated fomites of an infected individual [3]. Although the current outbreak disproportionately affects gay and bisexual men, evidence of sexual transmission via seminal or vaginal fluids has not yet been demonstrated [2]. Patients with the disease may exhibit systemic symptoms of fever, intense headache, lymphadenopathy, back pain, myalgia, and severe fatigue during the prodrome period for one to five days [3, 1]. Within one to three days of fever onset, a painful rash develops that evolves sequentially from macules to papules, vesicles, pustules, and finally dried crusts that shed from the skin [3, 1]. Its clinical presentation is similar to but notably less severe than that of the smallpox virus, another orthopoxvirus of renown due to its successful eradication worldwide in 1977, thanks in large part to coordinated global vaccination efforts [4]. On August 4, 2022, the United States Department of Health and Human Services declared the monkeypox outbreak a public health emergency. This report describes the first reported case of monkeypox infection in Calhoun County, Alabama, and its clinical course.

## Case presentation

A 52-year-old African American male presented to the emergency department with a one-week history of odynophagia to solids accompanied by pharyngeal and perianal pain without any other systemic symptoms. His past medical history was significant for prediabetes, neuropathy, hepatitis C, and HIV on treatment, but he was non-compliant with an undetectable viral load and a CD4 count of 303 cells/mm3. The patient’s medications included bictegravir (50 mg), emtricitabine (200 mg), and tenofovir alafenamide (25 mg) tablets daily, which he had stopped taking one month before admission. Further questioning revealed that the reason for the cessation of his medication was due to increased stress from moving recently and indifference to his condition. He had no significant family history. Social history was significant for a 20-pack smoking history per year and current tobacco and marijuana use. The patient received one Moderna COVID-19 vaccine and was up-to-date with influenza, pneumococcal, and tetanus vaccinations; however, he did not receive the smallpox vaccine. Three weeks before admission, with a one-month male partner, sexual history was significant for both insertive and receptive oral intercourse. The patient then had another sexual encounter with another male partner after one week, in which the two engaged in both insertive and receptive oral and anal intercourse. Both partners had been exposed to monkeypox by another male partner a few weeks before their encounters with the patient. The patient then developed painful perianal and pharyngeal vesicles one week after intercourse.

**Figure 1:**
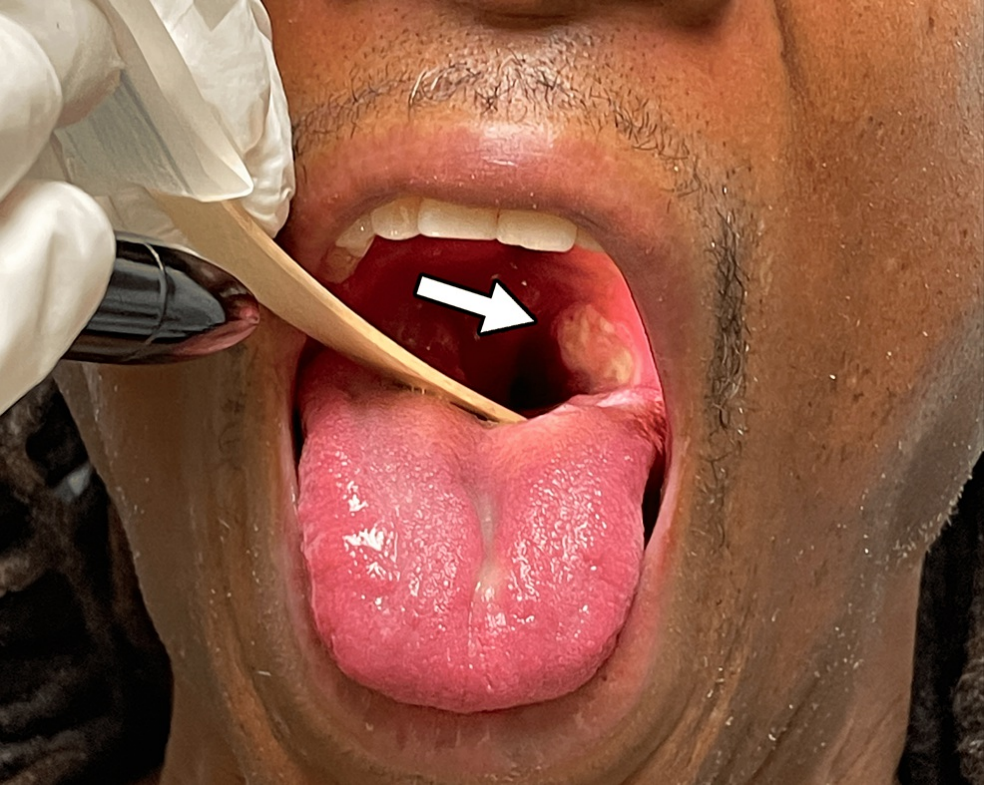
Left tonsillar hypertrophy with exudate

**Figure 2:**
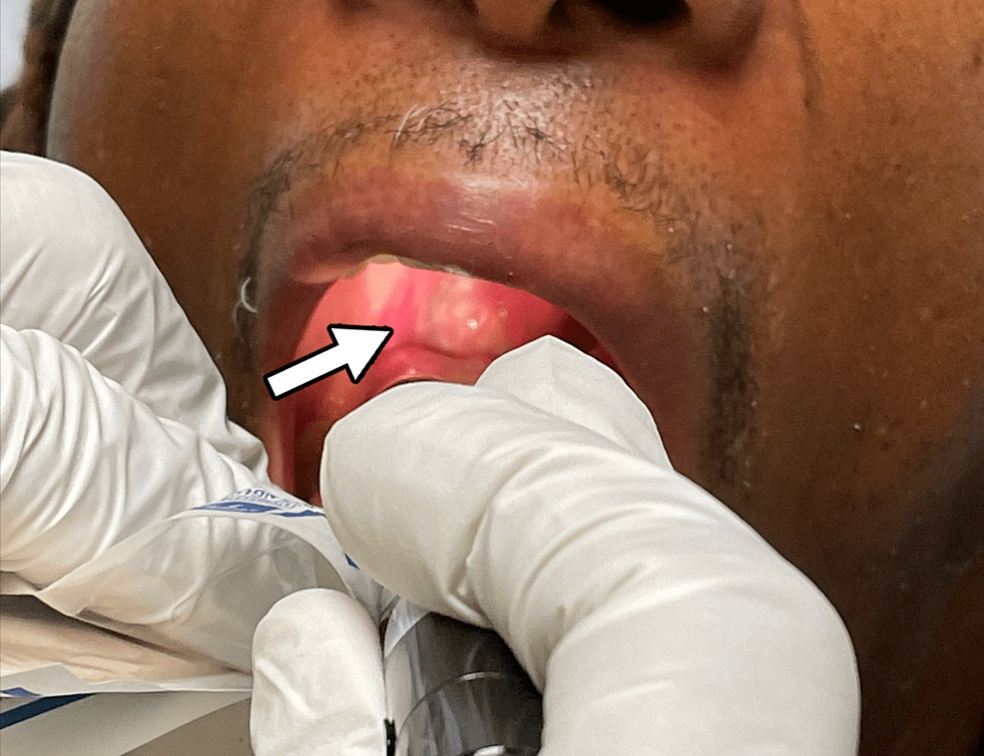
Right tonsillar hypertrophy with exudate

**Figure 3:**
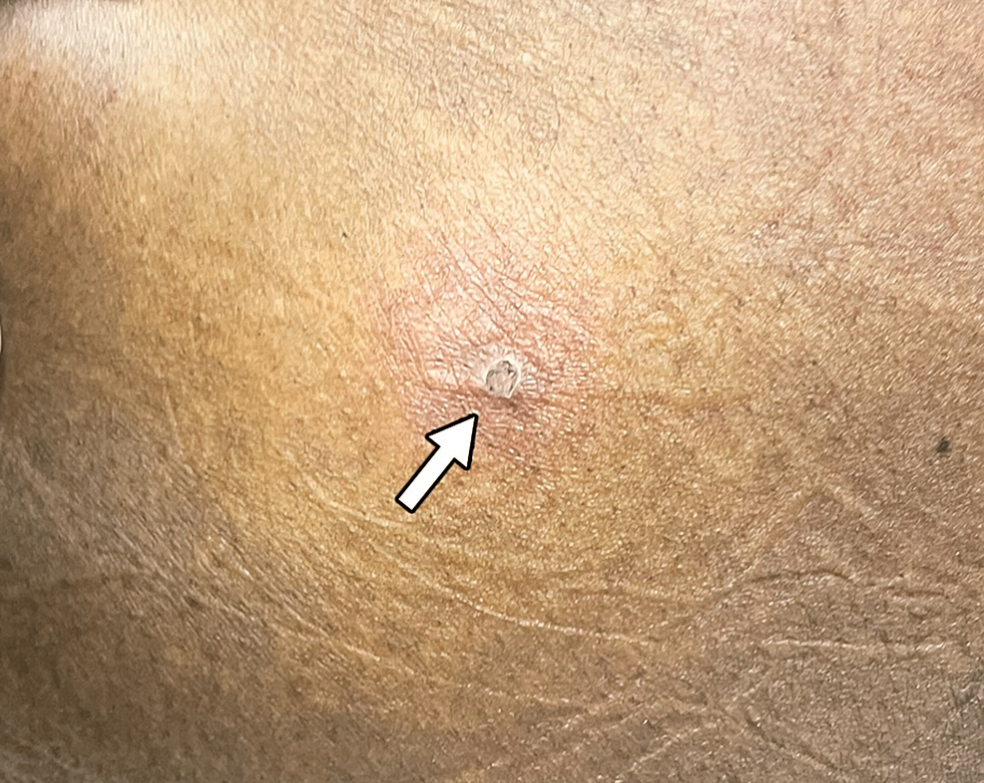
A monkeypox lesion on the right buttock

**Figure 4:**
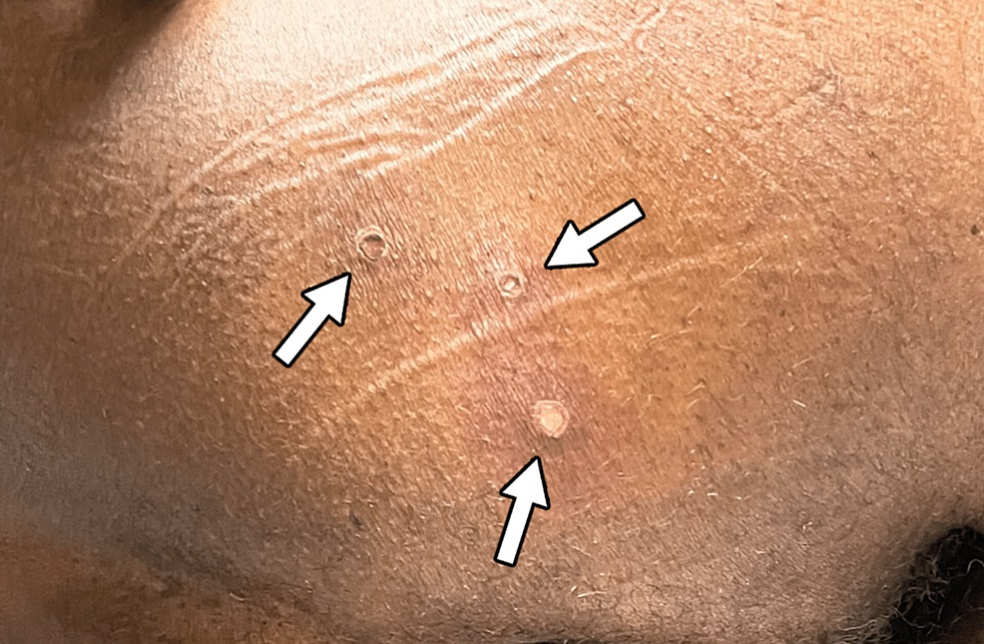
A cluster of monkeypox lesions on the right buttock

Physical examination revealed tonsillar hypertrophy with exudate (Figures 1, 2) and four 2-5 mm ulcerated lesions with overlying eschar on the right buttocks with surrounding erythema and tenderness to palpation (Figures 3, 4).

Computed tomography of the neck with contrast showed evidence of tonsillitis but no lymphadenopathy. Due to a high suspicion of monkeypox, the patient was placed under contact and airborne isolation precautions. Lab findings were significant for an elevated plasma reactive antibody titer of 1:16. Testing for Chlamydia trachomatis and Neisseria gonorrhoeae was negative. Monkeypox infection was confirmed with two positive polymerase chain reaction (PCR) tests to detect the DNA of orthopoxviruses, sampled from two separate lesions.

**Figure 5:**
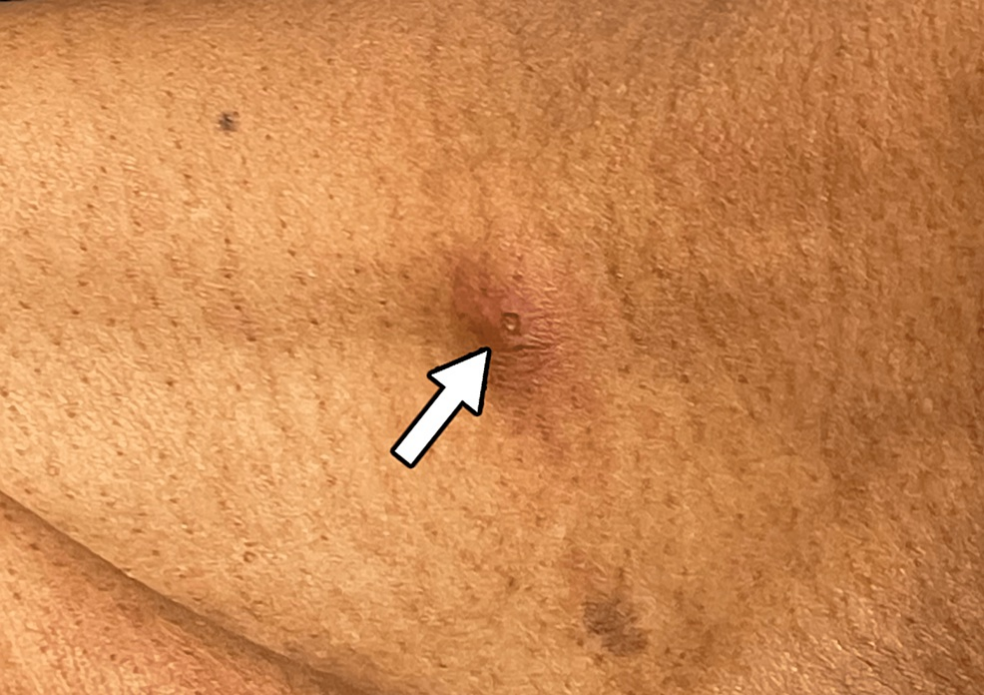
A monkeypox lesion above the right clavicle

**Figure 6:**
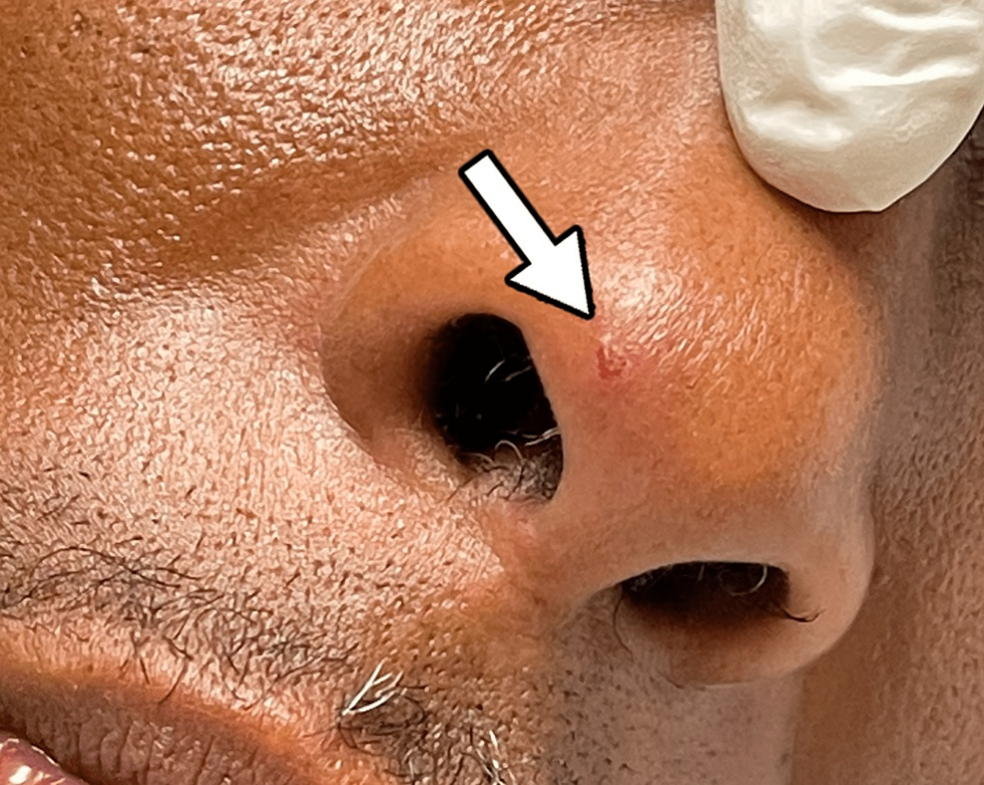
A monkeypox lesion on the tip of the nose

For syphilis, the patient was given penicillin G intramuscularly three times per week and was restarted on Biktarvy. A psychiatric evaluation was conducted to assess for depression and suicidal intent due to his history of sudden cessation of HIV medication. Throughout his hospital stay, the patient developed one new lesion above the right clavicle, one new lesion on the tip of his nose (Figures 5 and 6), and one macular lesion on the head of the penis. He also experienced the associated symptoms of fever or chills, asthenia, and diaphoresis, followed by pruritus at the tail end of the disease course. The patient is without signs of severe disease and has shown improvement with supportive care alone, without pharmacological treatment with tecovirimat.

## Discussion

Monkeypox is the most prominent Orthopoxvirus impacting humans following the eradication of smallpox [1]. To date, there have been 17,432 confirmed monkeypox cases in the United States, with 53 reported in the state of Alabama [3]. This case report is significant because it highlights the first reported case of monkeypox in Calhoun County, Alabama. This patient’s presentation was consistent with the expected course of mild, self-limiting monkeypox disease, and he had many notable risk factors for the disease in his history. The typical timeline of the illness generally begins with fever, followed by the development of multiple papular, vesiculopustular, and ulcerative lesions on the face and body and prominent lymphadenopathy. Complications include pneumonitis, encephalitis, keratitis, and secondary bacterial infections. Skin lesions were noted in 95% of the individuals. The most common anatomical sites were the anogenital area (73%); the trunk, arms, or legs (55%); the face (25%); and the palms and soles (10%). A wide spectrum of skin lesions was described, including macular, pustular, vesicular, and crusted lesions, and lesions in multiple phases were simultaneously present. Among persons with skin lesions, 58% had lesions that were described as "vesiculopustular" [2].

On presentation to the emergency department, this patient had a positive reactive plasma reagin titer of 1:16, raising initial suspicion for syphilis as the cause of his lesions. Only after a thorough history and physical exam was monkeypox added to the differential. Many people with monkeypox were initially misdiagnosed as having an STI due to their symptoms, prior medical history, and the vague rash that comes with monkeypox. A total of 54 people presented with only one genital ulcer, highlighting the possibility of misdiagnosis as a different STI. Concomitant STIs were reported in 109 of the 377 persons (29%) who were tested, with gonorrhea, chlamydia, and syphilis found in 8%, 5%, and 9%, respectively, of those who underwent testing [2].

HIV infection and being immunocompromised may or may not impact monkeypox presentation. The clinical presentation of people with HIV and those who did not have HIV was similar. Young children and immunocompromised persons, including persons living with HIV infection, have been reported to be at increased risk for severe outcomes, but whether effective antiretroviral therapy (ART) for HIV infection modifies this risk is unknown. [2]. Additionally, monkeypox reports on gay and bisexual men may show a disproportionate bias; although the current outbreak is disproportionately affecting gay or bisexual men, monkeypox is no more a "gay disease" than it is an "African disease." It can affect anyone. We identified nine heterosexual men with monkeypox [2].

Current treatment options include Tpoxx (tecovirimat). Tecovirimat shows antiviral activity via inhibition of viral spread from cell to cell via inhibition of the product of the F3L3 gene in orthopoxviruses. Animal model studies and clinical trials were utilized to gain FDA approval for tecovirimat as an effective treatment for smallpox under the Animal Rule. The Animal Rule has four requirements to satisfy: 1) The disease and the mechanism by which the countermeasure reduces or prevents it are both well understood; 2) countermeasure efficacy is demonstrated in one or multiple animal models that are considered to be well characterized and adequate for demonstration of efficacy; 3) efficacy endpoints in the animal model are related to the desired outcome in humans, such as improved survival or reductions in major morbidity; and 4) the human dose may be selected using data from animals treated at efficacious dose levels [5].

Tecovirimat was tested in several lethal challenge mouse modes, including both immunodeficient and immunocompetent mice. Results showed that treatment of orthopoxvirus-infected, immunocompromised mice resulted in prolonged survival as long as the drug was present, but when treatment was stopped, the disease reappeared and progressed to mortality. The drug was able to cure immunocompetent mice in that it slowed the spread of disease long enough for the host to mount an immune response and clear the virus. These results are consistent with its inhibitory effect on the virus through the interruption of the spread of disease rather than through the inhibition of replication or destruction of the virus [5].

To further examine the efficacy of tecovirimat, a series of cases of monkeypox treated by tecovirimat were examined and followed in Tecovirimat for the Treatment of Human Monkeypox: An Initial Series From Massachusetts, United States. The case presentation involved gay and bisexual men, two out of three being treated for HIV, who presented with a subset of symptoms including myalgia, rash, fever, ulcers, vesicles in areas of sexual contact, and odynophagia. Upon testing positive for monkeypox, patient one was admitted to the hospital for isolation and started on tecovirimat on day two of hospitalization-600 mg twice daily. By day four, new skin lesions ceased appearing, and existing skin lesions became less painful. By day six, this patient’s alanine transaminase (ALT) was elevated but had self-resolved by day eight; this patient was on no other systemic medications that would have caused a rise in ALT. Day 14 marked the end of therapy, and all symptoms had been resolved by this point in time. The only adverse effect reported by this patient was a mild, non-focal headache upon the start of therapy. Patient two followed a similar course, with all skin lesions crusting by Day 9 of therapy. This patient reported one or two loose bowel movements a few hours after each dose. Patient three received the same tecovirimat dosing schedule as patient two and experienced significant improvement and near-complete resolution of dermatologic changes by day seven of therapy. All completed the dose of tecovirimat, 14 days of 600 mg twice a day [6].

As a result of Tpoxx becoming approved by the CDC under the Investigational New Drug (EA-IND) protocol and available for use, the public health community now has an appropriate "countermeasure to treat patients in a smallpox emergency that previously had no recourse" [6]. Further studies performed during the development of tecovirimat show that the drug has great efficacy as a post-exposure prophylaxis therapeutic in the treatment of monkeypox.

Tecovirimat is currently available from the United States Strategic National Stockpile and can be administered under the careful monitoring specified under the CDC Institutional Review Board protocol. The CDC currently advises consideration of tecovirimat in patients with severe disease, those at risk for severe disease, and those with disease involvement in anatomic areas that might constitute a special hazard (such as the genitals) [6, 7]. As most patients may not be admitted to the hospital for care, further research should investigate more outpatient options. Alternative treatments are under investigation; however, serious adverse effects are still being researched. Brincidofovir, a nucleotide analog FDA-approved for treating smallpox, has demonstrated efficacy against MPXV in rodent models. Brincidofovir has decreased nephrotoxicity compared to its parent compound, cidofovir. However, all three patients treated with brincidofovir in the Liverpool outbreak discontinued therapy due to liver biochemical derangements [6, 8].

As a result of the current outbreak, further research into treatment options is urgently necessary. Large, well-controlled studies that are powered to demonstrate efficacy are urgently needed. Particular attention should be paid to any ability to accelerate the healing of lesions, as an agent with the potential to reduce the duration of infection would be particularly desirable [6].

Additionally, education and promotion of vaccination for pre-exposure prophylaxis and the prevention of severe clinical manifestations of monkeypox infection should be encouraged. JYNNEOSTM and ACAM2000® are two FDA-approved vaccines for the prevention of smallpox in high-risk individuals. Historical data have shown that smallpox vaccination with the vaccinia virus was approximately 85% effective against monkeypox [8]. Both JYNNEOSTM and ACAM2000® contain live vaccinia virus; however, the former is replication-deficient and the latter is replication-competent. Therefore, ACAM2000® is not recommended for use in immunosuppressed persons, such as those with HIV or those with an increased risk of unrecognized HIV, due to the risk of the development of progressive vaccinia [8].

## Conclusions

In summary, here we report a case of mild, self-limited monkeypox disease that did not require treatment with tecovirimat in an unvaccinated male patient with HIV, the first documented monkeypox case in Calhoun County, Alabama. As the current monkeypox outbreak continues to evolve, it is vital for healthcare professionals to be aware of this disease’s spread, clinical presentation, and conservative treatment.
